# Effectiveness of a Telehealth Intervention on Functional Status, Anxiety, Depression, and Rehospitalization Among Older Adults Undergoing Coronary Artery Bypass Grafting: Randomized Controlled Trial

**DOI:** 10.2196/81777

**Published:** 2026-05-07

**Authors:** Jirawan Mala, Usavadee Asdornwised, Kessiri Wongkongkam, Natkamol Chansatitporn, Punnarerk Thongcharoen

**Affiliations:** 1 Doctor of Philosophy Program in Nursing Science (International Program), Faculty of Medicine Ramathibodi Hospital and Faculty of Nursing, Mahidol University, Bangkok, Thailand; 2 Department of Surgical Nursing, Faculty of Nursing, Mahidol University, 2 Wang Lang Road, Siriraj, Bangkoknoi, Bangkok, 10700, Thailand, 66 2419746680, 66 24128415; 3 Faculty of Public Health, Mahidol University, Bangkok, Thailand; 4 Faculty of Medicine Siriraj Hospital, Mahidol University, Bangkok, Thailand

**Keywords:** telehealth, older adults, coronary artery bypass graft, transitional care model, functional status, anxiety, depression, rehospitalization

## Abstract

**Background:**

Telehealth has shown promise in enhancing care transitions and physical health outcomes in patients with cardiovascular disease. However, limited studies have explored its effect on functional status, psychological health, and rehospitalization, specifically in older patients undergoing coronary artery bypass grafting (CABG).

**Objective:**

This study aimed to evaluate the effectiveness of a telehealth intervention in improving functional status, reducing anxiety and depression, and decreasing rehospitalization rates compared with usual care among older patients undergoing CABG.

**Methods:**

The study was a 2-arm parallel randomized controlled trial. This was conducted in 2 phases. Phase 1 was conducted in the cardiac surgical units at a university hospital in Bangkok, Thailand. Phase 2 involved following up with the participant at home 30 and 90 days after discharge. A total of 84 older adults undergoing CABG were randomly assigned to either the control group (n=42), which received usual care (discharge planning), or the intervention group (n=42), which received a telehealth intervention based on the transitional care model in addition to usual care. The telehealth intervention included home monitoring via the “Zip Heart” app and scheduled video consultations. The primary outcome was functional status, measured using the Thai version of the Enforced Social Dependency Scale. Secondary outcomes included anxiety and depression, assessed using the Thai Hospital Anxiety and Depression Scale, and rates of rehospitalization. Data were collected at baseline, 30, and 90 days after discharge. Analyses were conducted using an intention-to-treat approach, with missing outcome data handled using multiple imputation. Two-way repeated-measures ANOVA was used to evaluate group, time, and group-by-time interaction effects.

**Results:**

A total of 84 participants were randomized and included in the intention-to-treat analysis (intervention group, n=42; control group, n=42). At baseline, there were no statistically significant differences between the two groups. Significant group-by-time interactions were observed for functional status scores (*F*_2,164_=32.09, *ηp*²=.28; *P<*.001), anxiety (*F*_2, 164_=20.22, *ηp²*=.2; *P<.*001), and depression (*F*_2,164_=16.81, *ηp²=*.17; *P<.*001). The intervention group demonstrated significantly greater improvements in functional status and greater reductions in anxiety and depression at both 30 and 90 days after discharge compared to the control group (all *P*<.001). Additionally, rehospitalization rates were significantly lower in the intervention group at 30 days (*Z*=2.77; *P*=.006) and between 31 and 90 days post discharge (*Z*=2.31; *P*=.02).

**Conclusions:**

The Telehealth intervention is effective in improving functional and psychological outcomes and reducing rehospitalization rates among older patients undergoing CABG. Integrating telehealth into usual care can support recovery and enhance continuity of care.

## Introduction

### Background

Coronary artery disease (CAD) is one of the leading causes of hospitalization and mortality worldwide, with older adults aged 60 years and older being the most affected [1]. Coronary artery bypass grafting (CABG) is the gold standard treatment for severe CAD and has been shown to improve survival, quality of life, and long-term cardiovascular outcomes by reducing recurrent myocardial infarction and the need for repeat revascularization [2,3]. This is particularly important given the increasing number of older patients requiring CABG, many of whom have multiple comorbidities, including hypertension, diabetes mellitus, and renal disease, which are common in older adults and require complex clinical management. Despite advancements in CABG, it remains highly stressful and traumatic for patients [4,5].

Older individuals undergoing CABG face many preoperative and postoperative physical and psychological challenges, which may adversely affect recovery and increase the risk of rehospitalization [4-8]. In Thailand, more than 6000 CABG procedures are performed annually [9], and most patients are older adults with preexisting comorbidities [10,11]. Rehospitalization within 28 days after discharge occurs in approximately 15.6% of patients and is commonly attributed to wound infections, fatigue, chest pain, and syncope [11].

Functional status is a key indicator of recovery after CABG, particularly in older patients. Older patients who underwent CABG have been reported to be particularly susceptible to decline in activities of daily living after surgery compared with their younger counterparts [12] and experience a slow recovery in functional status following surgery [6].

Psychological factors, particularly anxiety and depression, significantly influence recovery following CABG and are associated with delayed recovery, higher readmission rates, and increased mortality, especially in older adults [5,13]. These symptoms may persist or worsen after surgery due to the stress of surgery [4,5]. Preoperative anxiety and depression have been associated with higher mortality and poorer postoperative outcomes. Older patients undergoing CABG tend to experience higher levels of these symptoms than younger patients [5,14].

Inadequate postoperative care and ineffective communication may exacerbate psychological distress, resulting in poor care transitions and adverse outcomes [15,16]. System-level challenges, including workforce shortages, fragmented communication, and insufficient continuity of care, further contribute to care transition failures, increased adverse events, and higher rehospitalization rates [7,17-19]. Consequently, older adults with complex health conditions undergoing CABG are particularly vulnerable to poor postoperative outcomes.

Various programs, including standard discharge planning, cardiac rehabilitation, and patient education, are recommended to improve recovery and reduce rehospitalization after CABG [2]. Furthermore, nurses play a crucial role in guiding patients undergoing CABG and their family caregivers throughout the recovery process. However, usual care tends to be hospital-centered, with limited focus on home-based monitoring and postdischarge counseling.

### Telehealth

Telehealth has emerged as an important strategy for postdischarge management after CABG, particularly during the COVID-19 pandemic. Telehealth interventions have been shown to enhance patient engagement and satisfaction, support symptom monitoring, and enable timely clinical responses, thereby promoting continuity of care and reducing rehospitalization [11,20-30]. These interventions, including video consultations, remote monitoring, and educational platforms, also contribute to improvements in physical and psychological recovery.

Telehealth interventions, particularly those incorporating mobile health apps, have shown promise in improving functional health outcomes in older patients with cardiac conditions through cardiac rehabilitation and home monitoring [31,32]. Furthermore, telehealth, based on the transitional care model (TCM) integrating with remote monitoring and scheduled video visits, has also improved medication adherence, self-care, and rehospitalization outcomes in patients with chronic cardiovascular conditions [33,34]. However, most telehealth studies have been conducted in developed countries using diverse technologies and methods [35,36]. In Asia, telehealth has been applied to rehabilitation for long COVID-19 and home-based exercise among patients with cardiometabolic multimorbidity [37-39]. Nevertheless, evidence specific to older patients undergoing CABG remains limited, and prior studies in Thailand have largely focused on rehabilitation and monitoring, with less emphasis on psychological outcomes and postdischarge consultation [11,40]. Barriers to telehealth adoption among older adults include technological limitations and physical impairments [41,42]. Conversely, individualized approaches, patient-centered education, and age-friendly design features can enhance user satisfaction and engagement [43]. Furthermore, family and social support are critical for successful implementation of telehealth [44]. This study incorporated family caregivers into the telehealth intervention to address these challenges, enabling them to assist older patients undergoing CABG with daily activities and care management during the postdischarge period.

Therefore, the study aimed to evaluate the effectiveness of telehealth intervention in improving functional status, anxiety, depression, and rehospitalization compared with usual care among older patients undergoing CABG.

## Methods

### Overview

This study used a 2-arm, parallel-group, randomized controlled trial with a pretest-posttest control group design and repeated measures. Participants were randomly assigned to either the intervention or control group using a 1:1 block randomization method. The care protocols for each group are outlined below.

### Description of the Intervention

The intervention group received the telehealth intervention in addition to usual care. The telehealth intervention protocol was guided by the TCM [18] (Multimedia Appendix 1). The telehealth intervention was conducted by a researcher who was a specialist cardiac nurse with at least 5 years of experience in cardiac care. The care process started in the hospital and continued to the participants’ homes based on the eight core TCM components: (1) screening, (2) engaging the older adults and caregiver, (3) managing symptoms, (4) educating and promoting self-management, (5) collaboration, (6) assuring continuity, (7) coordinating care, and (8) maintaining relationships. The telehealth intervention encompassed 2 phases of the care process.

Phase 1 involved discharge planning at the cardiac surgical units. During hospitalization, the researcher developed individualized care plans, provided health education through a booklet, and trained participants and caregivers in self-management and the use of the “Zip Heart” app. Vital signs (eg, heart rate and blood pressure) were measured using automated devices and manually entered into the app by participants or caregivers. Devices and internet access were provided as needed, and follow-up video calls were scheduled prior to discharge.

**Figure 1.**
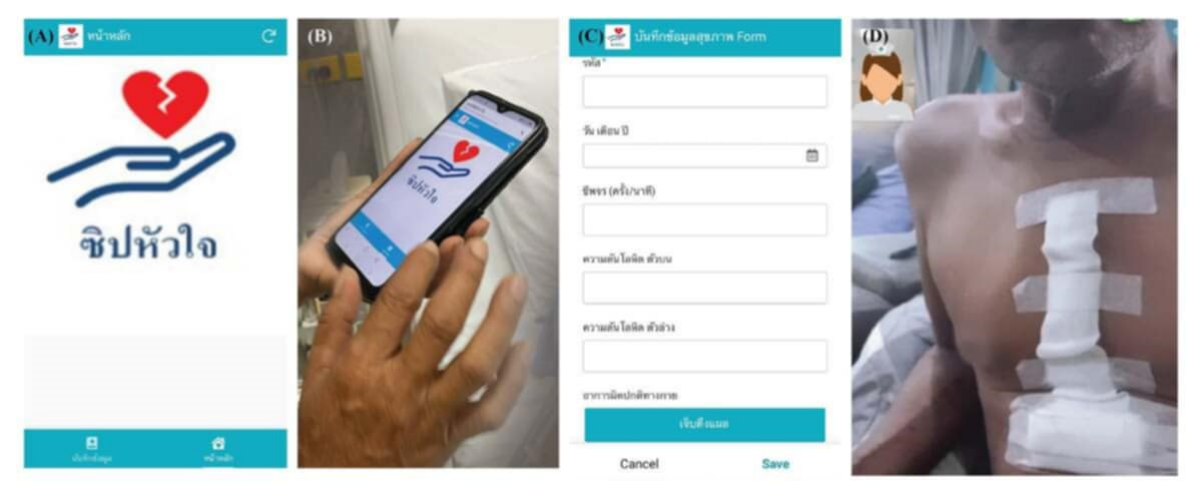
Representative telehealth intervention components: (A) Zip Heart app interface; (B) Zip Heart mobile app installation; (C) home monitoring; and (D) video call visit.

Phase 2 involved home monitoring via the “Zip Heart” app and video call visits, providing continuous postdischarge support and monitoring in the home setting. In the first month, the participants used the “Zip Heart” app to record their vital signs. They also received daily reminders and weekly video calls addressing symptoms, activities, and concerns, starting in the first week after discharge. Each call lasted 10‐20 minutes, depending on the participant’s recovery progress [25,45]. Video calls also enabled visual assessment of the participant’s physical condition and environment. The researcher consulted with a multidisciplinary team to address specific needs. All relevant data were recorded in the integrated discharge planning screening and follow-up form. Furthermore, infographics on postoperative care and cardiac rehabilitation were shared weekly. In the second and third months, the participants received biweekly video calls and weekly infographics on secondary cardiac prevention and behavior modification. Although all participants were scheduled for at least 8 video call visits during the study, the researcher used clinical judgment to determine if additional visits were needed. The video call visits ended when the participant was able to resume daily activities and required no further support from the health care team. An interview guide developed for this study was used to minimize respondents’ burden and avoid repetitive questioning. Participants in the intervention group continued to receive routine postoperative outpatient clinic follow-up as part of usual care. A representative telehealth intervention is presented in Figure 1.

### Control Group

The control group received usual care by the cardiac nurse, including orientation to the surgical unit, health education, and standard discharge planning. Postoperatively, the cardiac nurse and a multidisciplinary team monitored recovery, managed pain, encouraged rehabilitation exercises, and prepared participants and caregivers for discharge. On the day of discharge, participants received standardized discharge instructions based on the D-METHOD framework (Diagnosis, Medications, Environment, Treatment, Health behaviors, Outpatient follow-up, and Diet). This framework included information on diagnosis, medications, home environment, treatment regimen, health behaviors, outpatient follow-up, dietary recommendations, wound care, and potential complications. Participants were advised to attend a follow-up appointment with their cardiac surgeon one week after discharge at the outpatient department (OPD) and were provided with the surgical unit’s contact information for any health-related inquiries [11,46].

### Sample Size Calculation

The sample size was calculated using a formula recommended for experimental studies with repeated measures [47], with a significance level of .05 and a statistical power of 0.8. Functional status was selected as the primary outcome for this calculation. The estimation was informed by a previous study [11], which shared similar characteristics with this study and examined the effects of telehealth programs on functional status and rehospitalization rates among patients following CABG. In that study, functional status was measured using the Thai version of the Enforced Social Dependency Scale (Thai-ESDS) [48]. The SD of functional status scores in the population has been reported to range from 6 to 8.1. Four weeks after discharge, the experimental group had a mean functional status score of 13.59 (SD 3.35), compared to 17 (SD 5.98) in the control group, yielding a mean difference of 3.41. The corresponding effect size, calculated using Cohen d based on the group means and SDs, was 0.7, indicating a medium effect. Based on this effect size, the required sample size was estimated to be 33 participants per group. To account for anticipated attrition, the sample size was increased to 42 participants per group (84 in total), corresponding to an approximate attrition allowance of 21.4%.

### Sampling

A probability sampling method was used in this study. Participants were randomly selected from patients aged 60 years and older who were admitted for their first elective isolated CABG and met the inclusion criteria. Eligible participants were identified through the hospital’s surgical scheduling system, which served as the sampling frame. Patients were consecutively selected based on their scheduled surgery dates, according to the standard CABG surgical list, until the required sample size was reached.

### Participant Recruitment and Enrollment

All patients admitted for first-time elective isolated CABG were approached by nurses in the Cardiac Surgical Units on the day of hospital admission. These patients were provided with detailed information about the study. Individuals who expressed interest in participating were subsequently assessed by the researcher to determine eligibility based on predefined inclusion and exclusion criteria. Eligible individuals were invited to participate and were provided with a participant information sheet. Sufficient time was given to address any questions before obtaining written informed consent.

### Baseline Assessment

After providing written informed consent, all participants were assessed for baseline data by the researcher on the day of hospital admission. The assessments included (1) demographic and clinical characteristics, collected using the Health-Related Data Questionnaire (HRDQ); (2) functional status, measured using the Thai ESDS; and (3) levels of anxiety and depression, assessed using the Thai version of the Hospital Anxiety and Depression Scale (Thai HADS).

### Randomization and Allocation

Participants were randomly assigned to the intervention or control group (n=42 per group) using 1:1 block randomization to ensure balanced group sizes. The randomization sequence was generated using a computerized random number generator with a permuted block size of four in Microsoft Excel, with codes “A” (control) and “B” (intervention). Allocation concealment was maintained using sequentially numbered, opaque, sealed envelopes prepared according to the randomization sequence. Envelopes were opened only after informed consent was obtained. Participant allocation details, including serial number and patient identification codes, were recorded by the investigator and stored securely.

### Blinding Design

Blinding of treatment allocation was not feasible for either the nurses delivering the intervention or the participants due to the nature of the study design. Therefore, the trial was conducted using an open-label design. To minimize assessment bias, outcome data were collected by a research assistant who was blinded to group allocation, thereby ensuring observer blinding during data collection. However, a residual risk of unblinding remained, as participants may have inadvertently disclosed their group allocation during interviews.

### Study Design

This study was a single-center RCT with a pretest-posttest control group design and repeated measures. The trial evaluated the effectiveness of the telehealth intervention in addition to usual care, compared to usual care only, among older adult patients. This study was conducted in accordance with CONSORT (Consolidated Standards of Reporting Trials; checklist provided in Checklist 1) guidelines for randomized trials involving 2 groups[49] .

### Study Setting and Population

The study was conducted in 2 phases. Phase 1 took place in the cardiac surgical units of a university hospital in Bangkok, Thailand, between September 2023 and June 2024. Phase 2 involved home-based follow-up assessments at 30 and 90 days after discharge. The target population consisted of older adults undergoing CABG. All patients diagnosed with CAD who were scheduled for elective CABG and met the inclusion criteria were eligible and subsequently enrolled in the study.

### Inclusion and Exclusion Criteria

The inclusion criteria were patients aged 60 years or older and scheduled for first-time elective isolated CABG, those classified as functional class I-III according to the New York Heart Association (NYHA) classification [50], those able to access and use a smartphone with an Internet connection, either independently or with assistance from their primary family caregiver, those able to communicate in Thai, those without cognitive impairment, defined as a score of ≥3 on the Thai version of the Mini-Cog [51], and those having a primary family caregiver willing to participate in the study. The exclusion criteria were patients with severe dementia, Alzheimer disease, delirium, terminal illness, or psychiatric disorders diagnosed by a physician. Given the potential for physical or sensory impairments, activity limitations, and increased fall risk among older patients undergoing CABG, their primary family caregivers were included in the intervention to assist with daily activities.

Participants did not withdraw from the study; instead, some participants discontinued the telehealth intervention based on predefined criteria, including admission to a nursing home, refusal to continue participation after enrollment, failure to report physical measures or symptoms for five consecutive days with unsuccessful telephone contact for 3 consecutive days, intraoperative cardiac arrest, or severe postoperative complications such as stroke or renal failure requiring dialysis. All participants were retained in the final analysis.

### Treatment Fidelity

The telehealth intervention protocol was validated by a panel of cardiovascular experts to ensure treatment fidelity. The intervention was delivered by the researcher, a specialist cardiac nurse with over 5 years of experience in cardiovascular care, in collaboration with a multidisciplinary team. To maintain adherence to the intervention protocol and ensure quality control, the researcher received weekly supervision from her academic advisor throughout the study. A research assistant was a cardiac nurse who was independent of the intervention. She was trained to collect data at 30 and 90 days after discharge, and she was supervised during the data collection process by the researcher to prevent bias. Additionally, a pilot study was conducted prior to the main data collection to assess the feasibility and acceptability of the intervention; the pilot data were not published and were not included in the main analysis.

### Measurement

Four instruments were used for data collection: the HRDQ, the Thai ESDS, the Thai HADS, and the Number of Rehospitalizations Questionnaire. All instruments were validated by three clinical experts using the Content Validity Index (CVI) based on expert ratings of item relevance. Furthermore, Cronbach α coefficient was calculated using IBM SPSS Statistics version 18 to assess the instruments’ reliability. The participants were provided with standardized instructions on how to complete the questionnaires.

### HRDQ

The researcher developed the HRDQ to collect demographic characteristics, including age, gender, marital status, and education, and health-related information at baseline, such as CABG type, operative time, NYHA classification, and hospital length of stay (LOS). The CVI of the HRDQ was 1. Internal consistency reliability was not assessed because the items are descriptive and non-scaled.

### Thai ESDS

The ESDS was originally developed by Benoliel [52], refined for use in patients with chronic disease by McCorkle et al [53], and translated into Thai by Asdornwised [48]. The Thai ESDS measures changes in the functional status of patients with various illnesses, including cancer, CAD, and stroke, by evaluating performance before and after illness. The Thai ESDS consists of 10 items covering two domains: personal and social competence. The total social competence score ranges from 4 to 15, whereas the total functional status score ranges from 10 to 51, with higher scores indicating greater enforced dependency. Functional status scores are categorized as follows: 10‐23 indicates high independence, 24‐37 indicates moderate independence, and 38‐51 indicates low independence. The reliability score of the Thai ESDS was 0.71. The internal reliability (Cronbach α) was 0.80.

### Thai Hospital Anxiety and Depression Scale

The Hospital Anxiety and Depression Scale (HADS) was originally developed by Zigmond and Snaith [54] and was translated into Thai by Nilchaikovit [55]. The HADS assesses both anxiety and depression levels. The HADS consists of 14 items divided equally into two subscales: 7 items for anxiety and 7 items for depression. Each item is rated on a 7-point scale ranging from 0 to 3, with a total score of 0‐21 for each subscale. Scores on the Hospital Anxiety and Depression Scale–Anxiety subscale (HADS-A) are categorized as follows: 0‐7 indicates no anxiety, 8‐10 indicates mild anxiety, and 11‐14 indicates moderate anxiety. Similarly, scores on the Hospital Anxiety and Depression Scale–Depression subscale (HADS-D) are categorized as follows: 0‐7 indicates no depression, 8‐10 indicates mild depression, and 11‐14 indicates moderate depression. The Cronbach α coefficient for the HADS in Thai patients with CAD was 0.83 [40], whereas the Cronbach α for the anxiety and depression subscales was 0.70 in this study. A cut-off score of ≥8 was used to identify elevated anxiety and depression, consistent with validation studies in Thai patients with CAD [40].

### Number of Rehospitalizations Questionnaire

The researcher developed the Number of Rehospitalizations Questionnaire to record unplanned rehospitalizations due to cardiac, pulmonary, or surgery-related causes without prior appointments among participants. The questionnaire consisted of two sections: unplanned hospital readmissions and acute revisit to the OPD or emergency department (ED). The CVI of the questionnaire was 1, and reliability testing was not applicable due to its event-based nature.

### Data Collection

Functional status was defined as the primary outcome, with the 90-day follow-up serving as the primary endpoint, and the 30-day follow-up was included to evaluate early postoperative recovery. Anxiety, depression, and rehospitalization were assessed as secondary outcomes. Demographic and health-related data were collected using the HRDQ. Functional status was assessed using the Thai ESDS, and anxiety and depression were measured using the Thai HADS. The researcher collected baseline data on the day of admission to the Cardiac Surgical Units. Functional status, anxiety, depression, and rehospitalization were assessed 30 and 90 days after discharge or upon completion of the study. A research assistant who was blinded to group allocation collected the data via telephone interviews.

### Analytical and Statistical Approaches

Data were analyzed using IBM SPSS Statistics (version 18; IBM Corp). Descriptive statistics were used to summarize demographic characteristics, health-related variables, and baseline outcomes. Prior to group comparisons of continuous (ratio-scale) variables, the assumption of normality was assessed using the Kolmogorov–Smirnov test. Categorical variables were compared using chi-square tests, while continuous variables were analyzed using 2-tailed independent *t* tests. When assumptions for the chi-square test were not met, Fisher exact test was used as an alternative.

The impact of the telehealth intervention on functional status, anxiety, and depression over time was evaluated using a 2-way repeated-measures ANOVA. This analysis considered two factors: group (intervention vs control) and time (baseline [T1], 30 days [T2], and 90 days [T3] after discharge). Repeated-measures ANOVA was selected to reduce the risk of type I error. The necessary assumptions for this analysis, including sphericity and homogeneity of variances, were verified prior to conducting the tests.

All analyses were conducted according to the intention-to-treat (ITT) principle, whereby all randomized participants were analyzed in the groups to which they were originally assigned, regardless of adherence to the intervention. Participants who discontinued the telehealth intervention (eg, failure to report measurements for five consecutive days or the occurrence of severe intraoperative or postoperative complications) were retained in the analysis in accordance with the ITT framework. Missing data for continuous outcome variables were addressed using multiple imputation with fully conditional specification. Assuming the data were missing at random, five imputed datasets were generated. Each dataset was analyzed separately, and the results were pooled using Rubin’s rules to obtain the final estimates.

Differences in the proportions of participants who experienced rehospitalization due to cardiac, pulmonary, or surgery-related causes at 30 and 90 days after discharge between the intervention and control groups were evaluated using a 2-proportion *z* test. Rehospitalization was treated as a binary outcome at the participant level (event vs. no event) at each follow-up time point. Because no participants experienced repeated rehospitalization events during the follow-up period, count-based models (eg, Poisson regression) were not applicable. Therefore, a proportion-based approach using the 2-proportion *z* test was used to compare the proportion of participants experiencing rehospitalization between the intervention and control groups.

Percentages were calculated using the original randomized sample size as the denominator (n=42 per group) in accordance with the ITT principle. Participants who were lost to follow-up had no documented rehospitalization events during the monitoring period; therefore, consistent with a conservative ITT approach, these participants were classified as having experienced no rehospitalization events (coded as 0) in the analysis. Subcategories of rehospitalization events (unplanned readmission and acute care visits) were reported descriptively to illustrate the distribution of events and were not subjected to separate statistical testing. All statistical tests were two-tailed, and statistical significance was set at *P*<.05.

### Ethical Considerations

The study was reviewed and approved by the Institutional Review Board of Mahidol University (MU-MOU CoA No.IRB-NS2023/787.1807). The study was prospectively registered with the Thai Clinical Trials Registry (TCTR20230816008). All participants were provided with detailed information about the study, including the objectives, procedures, risks, benefits, confidentiality, and their right to withdraw without affecting their care. Written informed consent was obtained from all individuals prior to participation. To ensure privacy and confidentiality, all personal identifiers were removed and replaced with coded identification numbers. All data were stored securely and handled in accordance with institutional and ethical guidelines.

## Results

**Figure 2.**
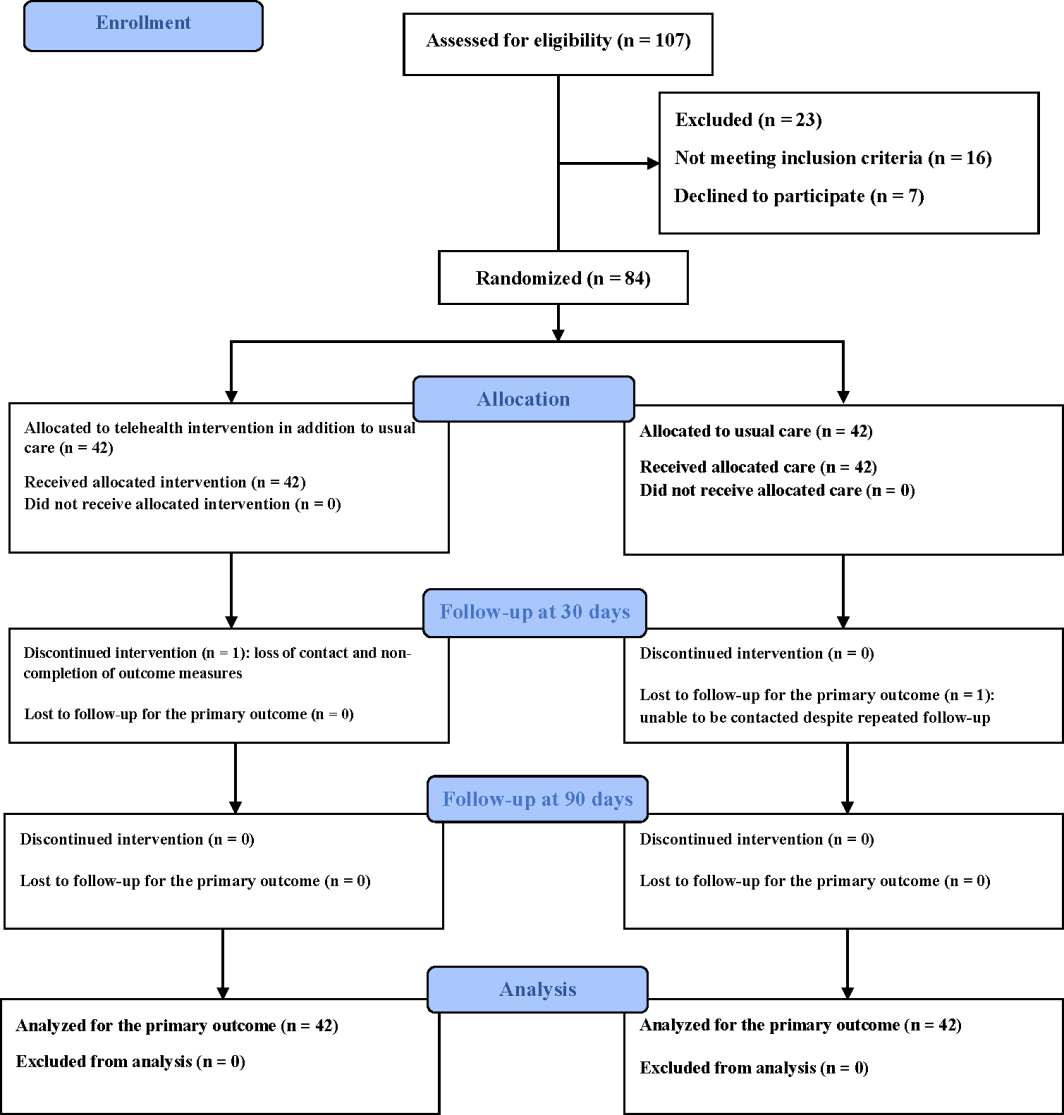
The process of the study according to the CONSORT (Consolidated Standards of Reporting Trials) flow diagram statistical analysis.

The flow of participants through the study is presented in Figure 2. The baseline demographic and health-related characteristics of the participants were comparable between the control and intervention groups (*P*>.05; see Table 1). The mean age of the participants was 67.07 (SD 6.56) years in the intervention group and 68.86 (SD 6.83) years in the control group. Most participants in both groups were male, married, and had primary school education. Most patients were diagnosed with triple-vessel disease and had comorbidities, including hypertension, diabetes mellitus (DM), and dyslipidemia, classified as NYHA class II, and underwent on-pump CABG surgery. The average LOS in the intensive care unit was 40.36 (SD 50.42) hours in the intervention group and 47.78 (SD 40.93) hours in the control group. Similarly, the mean hospital LOS was 9.64 (SD 3.91) days in the intervention group and 11.33 (SD 6.25) days in the control group.

**Table 1.** Baseline demographic and health-related characteristics of participants (n=84).

Characteristics	Intervention (n=42)	Control (n=42)	*P* value
Age (years), mean (SD)	67.07 (6.56)	68.86 (6.83)	.23^a^
Male, n (%)	30 (71.4)	28 (66.7)	.64^b^
Married, n (%)	36 (85.7)	31 (73.8)	.31^b^
Primary school education, n (%)	18 (42.9)	25 (59.5)	.36^b^
Type of CAD^d^, n (%)			.53^c^
DVD^e^	2 (4.8)	0 (0)	
TVD^f^	25 (59.5)	24 (57.1)	
TVD+ LM^g^	12 (28.6)	16 (38.1)	
DVD+ LM^h^	3 (7.1)	2 (4.8)	
Comorbidities, n (%)	40 (95.2)	41 (97.6)	.56^b^
HT^i^	35 (83.3)	38 (90.5)	
DM^j^	21 (50.0)	29 (69.0)	
DLP^k^	31 (73.8)	21 (50.0)	
CKD^l^	6 (14.3)	11 (26.2)	
≥2 comorbidities^m^	33 (78.6)	37 (88.1)	
NYHA^n^ functional classification, n (%)			.56^b^
II	33 (78.6)	36 (85.7)	
III	9 (21.4)	6 (14.3)	
Type of CABG^o^, n (%)			.31^b^
On-pump	39 (92.9)	41 (97.6)	
Off-pump	3 (7.1)	1 (2.4)	
Mean ICU^p^ LOS^q^, hours, mean (SD)	40.36 (50.42)	47.78 (40.93)	.46^a^
Mean Hospital LOS^q^, days, mean (SD)	9.64 (3.91)	11.33 (6.25)	.14^a^

a Independent *t* test.

b Chi-square test.

c Fisher exact test.

d CAD: coronary artery disease.

e DVD: double-vessel disease.

f TVD: triple-vessel disease.

g TVD + LM: triple-vessel disease with left main.

h DVD + LM: double-vessel disease with left main.

i HT: hypertension.

j DM: diabetes mellitus.

k DLP: dyslipidemia.

l CKD: chronic kidney disease.

m ≥2 comorbidities indicates the presence of two or more comorbid conditions (participants may have more than one comorbidity; therefore, percentages for individual conditions may exceed 100%).

n NYHA: New York Heart Association.

o CABG: coronary artery bypass grafting.

p ICU: intensive care unit.

q LOS: length of stay.

The 2-way repeated-measures ANOVA revealed a significant interaction between group and time for functional status scores (*F*_2, 164_=32.09, *ηp²*=.28; *P*<.001), anxiety (*F*_2, 164_=20.22, *ηp²*=.20; *P*<.001), and depression (*F*_2, 164_=16.81, *ηp²=*.17; *P*<.001), indicating that changes in mean scores for functional status, anxiety, and depression over time differed significantly between the intervention and control groups. Pairwise comparisons revealed significantly greater reductions in functional status scores across all time points in the intervention group than in the control group, indicating a steady improvement in functional independence. Additionally, significantly greater reductions in anxiety and depression scores were observed 30 and 90 days after discharge in the intervention group than in the control group (Table 2).

**Table 2.** Comparison of the differences in the mean scores for the study variables (n=84).

Variables and time	Intervention (n=42), mean (SD)	Control (n=42), mean (SD)	Group *F* (*P* value)	Time *F* (*P* value)	Group × time interaction *F* (*P* value)	95% CI^a^
Functional status^b^						
Baseline	23.02 (4.19)	21.17 (5.05)	15.53 (<.001)	37.63^c^ (<.001)	32.09^c^ (<.001)	−0.16 to 3.87
30 days	17.82 (3.82)	24.11 (6.58)	—^d^	—	—	−8.62 to −3.95
90 days	13.78 (3.12)	20.46 (7.84)	—	—	—	−9.28 to −4.10
Anxiety						
Baseline	7.94 (2.10)	7.97 (2.35)	52.17 (<.001)	84.25^c^ (<.001)	20.22^c^ (<.001)	−0.99 to 0.94
30 days	3.85 (1.64)	6.65 (2.87)	—	—	—	−3.81 to −1.78
90 days	1.48 (0.86)	5.74 (3.33)	—	—	—	−5.32 to −3.21
Depression						
Baseline	4.28 (1.79)	4.17 (1.77)	19.47 (<.001)	32.96^c^ (<.001)	16.81^c^ (<.001)	−0.67 to 0.88
30 days	2.78 (1.46)	4.38 (2.70)	—	—	—	−2.54 to −0.66
90 days	0.85 (0.94)	3.63 (2.62)	—	—	—	−3.63 to −1.92

a 95% CI of the between-group mean difference at each time point.

b Lower scores of the mean scores of functional status reflect higher functional independence.

c Repeated-measures ANOVA sphericity assumed.

d Not available.

Rehospitalizations were defined as any unplanned hospital-based use occurring within 30 or 90 days after discharge, including (1) unplanned inpatient readmissions and (2) acute visits to the OPD or ED for cardiac, pulmonary, or surgery-related issues.

Within 30 days after discharge, rehospitalizations occurred significantly more frequently in the control group than in the intervention group (16/42, 38.1%) vs (5/, 11.9%); Z=2.77; *P*=.006). Similarly, between 31 and 90 days post discharge, rehospitalizations remained higher in the control group (5/42, 11.9%) compared with the intervention group (n=0, 0%), with a statistically significant between-group difference (Z=2.31; *P*=.02). Overall, participants in the intervention group experienced significantly fewer rehospitalization events than those in the control group during both follow-up intervals (Table 3).

**Table 3.** Comparison of rehospitalization events between the intervention and control groups during the 90-day follow-up period after discharge (n=84).

Variables	Intervention (n=42), n (%)^a^	Control (n=42), n (%)^a^	*Z* test^b^	*P* value
Rehospitalizations within 30 days^a^	5 (11.9)	16 (38.1)	2.77	.006
Unplanned readmission^c^	0 (0)	9 (21.4)	—^e^	—
Acute care visits^c^	5 (11.9)	7 (16.7)	—	—
Rehospitalizations between 31 and 90 days postdischarge^d^	0 (0)	5 (11.9)	2.31	.02
Unplanned readmission^c^	0 (0)	4 (9.5)	—	—
Acute care visits^c^	0 (0)	1 (2.4)	—	—

a Data are presented as n (%). Percentages were calculated using the intention-to-treat denominator (n=42 per group).

b Between-group comparisons of overall rehospitalization proportions within each time interval were conducted using the two-proportion *z* test.

c Subcategories of rehospitalization events (unplanned readmission and acute care visits) are presented for descriptive purposes only; no statistical comparisons were performed for these subcategories.

d Rehospitalization counts represent observed events occurring during the follow-up period after hospital discharge.

e Not available.

At 30 days after discharge, causes of unplanned hospital readmissions in the control group included heart failure (n=2), septic shock (n=2), wound infection (n=2), fatigue (n=1), hypotension (n=1), and pleural effusion (n=1). No unplanned hospital readmissions occurred in the intervention group during this period. Acute visits to the OPD or ED were also more frequent in the control group, with documented causes including hypotension (n=2), sternal wound bleeding (n=1), wound infection (n=1), severe hypertension (n=1), tiredness (n=1), and localized leg edema (n=1). In the intervention group, acute visits occurred due to wound infection (n=4) and severe pain (n=1); however, none resulted in hospital readmission.

During the 31‐90 days post discharge, unplanned hospital readmissions in the control group were attributed to heart failure (n=2), dyspnea (n=1), and wound infection (n=1). No unplanned hospital readmissions were recorded in the intervention group during this period. An acute OPD visit due to dyspnea (n=1) was documented in the control group, whereas no acute visits or rehospitalizations were observed in the intervention group. No adverse events related to the intervention were identified. Furthermore, no deaths occurred in either group during the 90-day follow-up period.

## Discussion

### Principal Findings

The findings of this study demonstrate the potential of a telehealth intervention, delivered through the “Zip Heart” app and video consultations, to improve functional recovery, reduce psychological symptoms, and lower rehospitalization rates among older adults undergoing CABG. Integrating telehealth into usual care appears to be a promising strategy for providing continuous support during the critical postdischarge period. The effectiveness of the intervention may be attributed to its foundation in the TCM [18], which emphasizes continuity, coordination, and sustained patient–nurse relationships—key elements for effective care management during transitions of care [17,56]. These findings are consistent with previous studies showing that TCM-based interventions reduce rehospitalization rates and health care costs while improving functional independence and quality of life in older patients following open-heart surgery [57-59].

The integration of the “Zip Heart” app with scheduled video consultations enabled effective remote monitoring and addressed both clinical and informational needs during the transition from hospital to home. Timely professional support and ongoing education facilitated a smoother recovery process. In line with these findings, prior research has shown that telehealth monitoring combined with video consultations enhances health literacy and self-management skills among older adults with chronic conditions [60], while web-based health education and telecounseling interventions are effective in alleviating postoperative psychological distress [26,61,62]. However, self-home monitoring may pose challenges for frail or vulnerable older adults due to technological barriers [28]. In this context, caregiver involvement plays a critical role in supporting recovery and facilitating successful telehealth implementation. A hybrid care model that combines telehealth with conventional in-person services may therefore be more appropriate for this population.

Although postoperative complications occurred in both study groups, the rehospitalization rate was lower in the intervention group. This suggests that the telehealth intervention may have supported earlier identification and management of postoperative issues, enabling timely outpatient treatment and reducing the need for hospital readmissions. Prompt responses to emerging complications may prevent their progression to more severe conditions requiring rehospitalization. However, rehospitalization risk is influenced by patient characteristics. In this study, participants were older adults aged 60‐88 years with a prevalence of comorbidities, including hypertension, DM, dyslipidemia, and chronic kidney disease. Socioeconomic factors, such as low educational attainment, unemployment, and limited income, may also have contributed to reduced health literacy and difficulty understanding postoperative care instructions [63]. In addition, the absence of continuous monitoring and regular health care provider contact in the control group may have delayed the recognition of early risk factors for rehospitalization.

The findings further underscore the expanding role of nurses in delivering digitally enabled, patient-centered care. With appropriate training, nurses can effectively coordinate and manage care through telehealth platforms, thereby strengthening continuity between hospital-based and home-based services. This positions nursing practice at the forefront of implementing innovative care models tailored to the needs of an aging cardiac population. However, the telehealth intervention in this study was delivered by a specialist cardiac nurse, which may have increased personnel burden and introduced therapeutic effects related to advanced clinical expertise. The requirement for delivery by a specialist cardiac nurse represents a significant limitation, as it may constrain the generalizability and reproducibility of the intervention in settings with limited access to specialized nursing staff. Additionally, telehealth interventions play a critical role in preventing complications that lead to rehospitalization; their effectiveness may vary depending on patient characteristics, targeted outcomes, multidisciplinary involvement [64], and intervention intensity or dosage [56]. Moreover, successful implementation requires careful consideration of associated costs, including investments in technological infrastructure, staff training, and workload management strategies. Addressing these factors would be essential to ensure the long-term sustainability and scalability of telehealth-supported transitional care programs.

### Limitations

Several limitations should be considered when interpreting these findings. First, this was a single-center study conducted in Thailand with a specific patient population, which may limit the generalizability of the findings to other health care settings and populations with different demographic or health system characteristics.

Second, the intervention relied on telehealth tools, including video consultations and the “Zip Heart” app. Although outcomes were assessed at 30 and 90 days post discharge, providing short- and midterm data, the long-term effects of the intervention on functional status, psychological outcomes, and rehospitalization remain unknown.

Third, the eligibility criteria required participants to have access to and be able to use a smartphone with an internet connection. This requirement may have excluded older adults with lower socioeconomic status or limited digital literacy, thereby restricting the generalizability of the findings to the broader older population.

Fourth, participants in the intervention group received more frequent contact with health care providers through scheduled video consultations compared with the control group. This increased attention may have introduced performance and attention bias (Hawthorne effect), potentially contributing to improvements in psychological outcomes. Although no participants required video consultations beyond those prespecified, the allowance for clinical discretion may still have introduced potential subjective bias. To minimize this risk, consultation frequency was aligned with the postoperative recovery trajectory, and a structured consultation guide was used to ensure consistency and reduce participant burden. In addition, the costs associated with technology implementation and staff training were not evaluated, limiting assessment of the cost-effectiveness and scalability of the intervention.

Fifth, rehospitalization data were obtained through participant or caregiver report and cross-checked against available medical records when possible. However, complete verification was not feasible for all cases due to data protection constraints, and some data relied on self-report, introducing potential recall bias. Moreover, rehospitalization was analyzed as a binary outcome, which does not capture the frequency or timing of recurrent events.

Sixth, although outcome data were collected by a blinded research assistant, the open-label design and reliance on patient-reported outcome measures may have introduced social desirability bias, potentially leading to overestimation of favorable outcomes.

Finally, 2 participants were lost to follow-up during the study period. One participant in the control group was lost to follow-up despite repeated contact attempts, and one participant in the intervention group failed to report outcome measures for five consecutive days and could not be reached by phone during the 30-day follow-up period. No additional attrition occurred during the subsequent 90-day follow-up period. The overall attrition rate was low (2.38%) and substantially lower than the anticipated attrition rate (21.4%). To maintain the integrity of randomization and minimize bias due to missing data, an ITT analysis was performed in which all randomized participants (n=84) were included in the analysis. Missing data for continuous outcomes were handled using multiple imputation, whereas a conservative zero-event assumption was applied to participants lost to follow-up for the binary outcome of rehospitalization.

### Future Research Directions

This study focused exclusively on older adults undergoing CABG surgery. Future research should include other patient populations with complex health care needs to enhance the generalizability of the findings. Investigating the implementation and effectiveness of telehealth across diverse clinical contexts can broaden our understanding of its applicability and scalability. Additionally, future studies should incorporate larger sample sizes and extended follow-up periods to capture long-term outcomes. Future investigations may also consider more advanced analytical approaches to examine rehospitalization outcomes in greater detail. For example, count-based regression models (eg, Poisson or negative binomial regression) could be used to analyze the frequency of rehospitalizations when repeated events occur, while survival analysis methods could evaluate the timing of readmissions.

The telehealth intervention used in this study integrated a mobile app and video consultations to support postdischarge care. Future studies should investigate the potential of emerging technologies, such as artificial intelligence–driven platforms, wearable health-monitoring devices, and virtual reality–based rehabilitation tools. Comparative effectiveness studies evaluating these different modalities could yield important insights into identifying the most effective strategies for optimizing recovery and improving outcomes among older adults undergoing CABG.

Finally, the economic viability of telehealth interventions should be rigorously assessed through cost-effectiveness analyses, particularly with regard to their potential to reduce health care use and improve long-term patient outcomes.

### Conclusions

This study demonstrated that telehealth intervention had significant positive effects on improving functional status, reducing anxiety and depression, and lowering rehospitalization rates among older patients undergoing CABG who received telehealth intervention in addition to usual care. Telehealth intervention facilitated continuous monitoring and timely support during the critical postdischarge period, thereby enhancing recovery and overall quality of care.

## Supplementary material

10.2196/81777 Multimedia Appendix 1 Theoretical framework.

10.2196/81777 Checklist 1 CONSORT checklist.

## Abbreviations

CABG: coronary artery bypass grafting
CAD: coronary artery disease
CONSORT: Consolidated Standards of Reporting Trials
CVI: content validity index
D-METHOD: diagnosis, medications, environment, treatment, health behaviors, outpatient follow-up, and diet
DM: diabetes mellitus
ED: emergency department
ESDS: Enforced Social Dependency Scale
HADS: Hospital Anxiety and Depression Scale
HADS-A: Hospital Anxiety and Depression scale–Anxiety Subscale
HADS-D: Hospital Anxiety and Depression Scale–Depression Subscale
HRDQ: Health-Related Data Questionnaire
ITT: intention-to-treat
LOS: length of stay
NYHA: New York Heart Association
OPD: outpatient department
TCM: transitional care model
