# Supplementary material for: Effectiveness of a Telehealth Intervention on Functional Status, Anxiety, Depression, and Rehospitalization Among Older Adults Undergoing Coronary Artery Bypass Grafting: Randomized Controlled Trial
Source: JMIR Cardio. 2026 May 7;10:e81777. doi: 10.2196/81777 (PMC13152204; doi:10.2196/81777)
Supplement: Multimedia Appendix 1 [file cardio-v10-e81777-s001.docx]

**Theoretical Framework**

A literature review of studies that used the Transitional Care Model (TCM) [18] to guide continuing care from hospitalization to home for older patients after CABG revealed a significant improvement in their quality of life and functional autonomy [20-22]. Additionally, this approach reduced rehospitalization and improved physical outcomes. The TCM is a nurse-led, multidisciplinary care management model designed to enhance care transitions, particularly for vulnerable populations. It was introduced as a framework for improving the quality of transitional care. The TCM was developed in 1981 at the University of Pennsylvania School of Nursing [18]. It has been extensively evaluated through randomized controlled trials in various populations, including mothers with diabetes and hypertension and hospitalized older patients with cardiac medical and surgical conditions. A transitional care nurse (TCN) is a nursing specialist with advanced expertise who plays a crucial role in the TCM framework. TCNs support older patients during their transition from hospital to home by providing evidence-based care tailored to the needs of patients and their family caregivers, thereby improving health outcomes and quality of care. Additionally, TCNs collaborate with a multidisciplinary team to address diverse health concerns during this critical period. The TCM incorporates eight core components: screening, engaging older patients and their caregivers, symptom management, education and self-management promotion, collaboration, continuity of care, care coordination, and relationship building. These interdependent components create an integrated and seamless continuum of care.
