# Supplementary material for: Effectiveness of a Telehealth Intervention on Functional Status, Anxiety, Depression, and Rehospitalization Among Older Adults Undergoing Coronary Artery Bypass Grafting: Randomized Controlled Trial
Source: JMIR Cardio. 2026 May 7;10:e81777. doi: 10.2196/81777 (PMC13152204; doi:10.2196/81777)
Supplement: Checklist 1 [file cardio-v10-e81777-s002.pdf]

Section/topic	No	CONSORT 2025 checklist item description	Reported on page no.
**Title and abstract**			
Title and structured abstract	1a	Identification as a randomised trial	1
	1b	Structured summary of the trial design, methods, results, and conclusions	1
**Open science**			
Trial registration	2	Name of trial registry, identifying number (with URL) and date of registration	2
Protocol and statistical analysis plan	3	Where the trial protocol and statistical analysis plan can be accessed	12
Data sharing	4	Where and how the individual de-identified participant data (including data dictionary), statistical code and any other materials can be accessed	7, 12
Funding and conflicts of interest	5a	Sources of funding and other support (eg, supply of drugs), and role of funders in the design, conduct, analysis and reporting of the trial	12
	5b	Financial and other conflicts of interest of the manuscript authors	12
**Introduction**			
Background and rationale	6	Scientific background and rationale	2
Objectives	7	Specific objectives related to benefits and harms	3
**Methods**			
Patient and public involvement	8	Details of patient or public involvement in the design, conduct and reporting of the trial	3-4
Trial design	9	Description of trial design including type of trial (eg, parallel group, crossover), allocation ratio, and framework (eg, superiority, equivalence, non-inferiority, exploratory)	4
Changes to trial protocol	10	Important changes to the trial after it commenced including any outcomes or analyses that were not prespecified, with reason	
Trial setting	11	Settings (eg, community, hospital) and locations (eg, countries, sites) where the trial was conducted	5
Eligibility criteria	12a	Eligibility criteria for participants	5
	12b	If applicable, eligibility criteria for sites and for individuals delivering the interventions (eg, surgeons, physiotherapists)	3
Intervention and comparator	13	Intervention and comparator with sufficient details to allow replication. If relevant, where additional materials describing the intervention and comparator (eg, intervention manual) can be accessed	3-4
Outcomes	14	Prespecified primary and secondary outcomes, including the specific measurement variable (eg, systolic blood pressure), analysis metric (eg, change from baseline, final value, time to event), method of aggregation (eg, median, proportion), and time point for each outcome	6
Harms	15	How harms were defined and assessed (eg, systematically, non-systematically)	7
Sample size	16a	How sample size was determined, including all assumptions supporting the sample size calculation	4
	16b	Explanation of any interim analyses and stopping guidelines	4
Randomisation:			
Sequence generation	17a	Who generated the random allocation sequence and the method used	5
	17b	Type of randomisation and details of any restriction (eg, stratification, blocking and block size)	5

			Reported on page no.
Allocation concealment mechanism	18	Mechanism used to implement the random allocation sequence (eg, central computer/telephone; sequentially numbered, opaque, sealed containers), describing any steps to conceal the sequence until interventions were assigned	5
Implementation	19	Whether the personnel who enrolled and those who assigned participants to the interventions had access to the random allocation sequence	5
Blinding	20a	Who was blinded after assignment to interventions (eg, participants, care providers, outcome assessors, data analysts)	5
	20b	If blinded, how blinding was achieved and description of the similarity of interventions	5
Statistical methods	21a	Statistical methods used to compare groups for primary and secondary outcomes, including harms	6
	21b	Definition of who is included in each analysis (eg, all randomised participants), and in which group	6
	21c	How missing data were handled in the analysis	6
	21d	Methods for any additional analyses (eg, subgroup and sensitivity analyses), distinguishing prespecified from post hoc	6
**Results**			
Participant flow, including flow diagram	22a	For each group, the numbers of participants who were randomly assigned, received intended intervention, and were analysed for the primary outcome	7
	22b	For each group, losses and exclusions after randomisation, together with reasons	7-8
Recruitment	23a	Dates defining the periods of recruitment and follow-up for outcomes of benefits and harms	7-8
	23b	If relevant, why the trial ended or was stopped	7-8
Intervention and comparator delivery	24a	Intervention and comparator as they were actually administered (eg, where appropriate, who delivered the intervention/comparator, how participants adhered, whether they were delivered as intended (fidelity))	3, 7-8
	24b	Concomitant care received during the trial for each group	3, 7-8
Baseline data	25	A table showing baseline demographic and clinical characteristics for each group	8
Numbers analysed, outcomes and estimation	26	For each primary and secondary outcome, by group:<br>● the number of participants included in the analysis<br>● the number of participants with available data at the outcome time point<br>● result for each group, and the estimated effect size and its precision (such as 95% confidence interval)<br>● for binary outcomes, presentation of both absolute and relative effect size	7-10
Harms	27	All harms or unintended events in each group	10
Ancillary analyses	28	Any other analyses performed, including subgroup and sensitivity analyses, distinguishing pre-specified from post hoc	10
**Discussion**			
Interpretation	29	Interpretation consistent with results, balancing benefits and harms, and considering other relevant evidence	10-11
Limitations	30	Trial limitations, addressing sources of potential bias, imprecision, generalisability, and, if relevant, multiplicity of analyses	11

Citation: Hopewell S, Chan AW, Collins GS, Hróbjartsson A, Moher D, Schulz KF, et al. CONSORT 2025 Statement: updated guideline for reporting randomised trials. BMJ. 2025; 388:e081123. https://dx.doi.org/10.1136/bmj-2024-081123
© 2025 Hopewell et al. This is an Open Access article distributed under the terms of the Creative Commons Attribution License (https://creativecommons.org/licenses/by/4.0/), which permits unrestricted use, distribution, and reproduction in any medium, provided the original work is properly cited.

*We strongly recommend reading this statement in conjunction with the CONSORT 2025 Explanation and Elaboration and/or the CONSORT 2025 Expanded Checklist for important clarifications on all the items. We also recommend reading relevant CONSORT extensions. See www.consort-spirit.org.
